# Wearable devices for patient monitoring in the intensive care unit

**DOI:** 10.1186/s40635-025-00738-8

**Published:** 2025-02-27

**Authors:** Alessandra Angelucci, Massimiliano Greco, Maurizio Cecconi, Andrea Aliverti

**Affiliations:** 1 Dipartimento di Elettronica, Informazione e Bioingegneria, Politecnico di Milano, Milan, Italy; 2 Department of Biomedical Sciences, Humanitas University, Pieve Emanuele, Italy; 3 Department of Anesthesiology and Intensive Care, IRCCS Humanitas Research Hospital, Rozzano, Italy

**Keywords:** Wearable devices, Intensive care unit, Continuous monitoring, Non-invasive technology, Critical care

## Abstract

Wearable devices (WDs), originally launched for fitness, are now increasingly recognized as valuable technologies in several clinical applications, including the intensive care unit (ICU). These devices allow for continuous, non-invasive monitoring of physiological parameters such as heart rate, respiratory rate, blood pressure, glucose levels, and posture and movement. WDs offer significant advantages in making monitoring less invasive and could help bridge gaps between ICUs and standard hospital wards, ensuring more effective transitioning to lower-level monitoring after discharge from the ICU. WDs are also promising tools in applications like delirium detection, vital signs monitoring in limited resource settings, and prevention of hospital-acquired pressure injuries. Despite the potential of WDs, challenges such as measurement accuracy, explainability of data processing algorithms, and actual integration into the clinical decision-making process persist. Further research is necessary to validate the effectiveness of WDs and to integrate them into clinical practice in critical care environments.

Take home messages

- Wearable devices are revolutionizing patient monitoring in ICUs and step down units by providing continuous, non-invasive, and cost-effective solutions.
- Validation of their accuracy and integration in the clinical decision-making process remain crucial for widespread clinical adoption.

## Introduction

Advances in technology have led to the development of innovative tools such as wearable devices (WDs), portable devices, and point-of-care devices, which are gaining increasing attention for their potential to enhance healthcare delivery. In this context, we focus on wearables, lightweight devices specifically designed to be worn non-invasively on the body. Even though some articles refer to WDs as “wearable biosensors”, it must be noted that not all sensors used in WDs are biosensors. Biosensors are specialized devices that integrate a biological element, such as enzymes or antibodies, with a physicochemical detector to measure biochemical changes, enabling the detection of specific biomarkers in the body. Only a few sensors qualify as biosensors; the majority detect changes that are not biochemical. Another common misconception in the literature is the interchangeable use of the terms 'wearable device' and 'wearable sensor.' A wearable sensor refers solely to the sensing component, whereas a wearable device encompasses additional electronic components, such as a microcontroller or an antenna for data transmission. For this reason, this paper will focus on WDs as a whole.

WDs have been originally developed as applications for monitoring vital signs during athletic activities, but soon the medical community discovered their usefulness for wireless remote monitoring of outpatients at low cost. WDs may be useful for hospitalized patients as well, especially those who are unstable or at higher risk for serious complications, such as critically ill patients in the intensive care unit (ICU) and step down units [1].

By means of WDs, it is possible to monitor vital signs (heart/pulse rate, blood pressure, respiratory rate, temperature [2]), glucose levels, body position, and activity, among other things. This type of technology allows for non-invasive frequent measurements, or continuous monitoring [3].

Patients in the ICU require extensive monitoring, which can be time-consuming and challenging due to the need to minimize false readings. In western countries, the swift detection of physiological anomalies and prompt intervention are achieved through advanced monitoring systems, supported by high nurse-to-patient ratios. In low-income and middle-income countries (LMIC), deficiency of personnel and equipment makes this monitoring more difficult to achieve, creating opportunities for low-cost wearable devices to provide a viable solution[4].

Wearables can help reduce medical errors, minimize the risk of adverse events, and save both time and costs [5]. For instance, Nherera et al. [6] published an economic evaluation to assess whether the use of the LEAF Patient Monitoring System™ (Smith + Nephew, Inc., Watford, UK) [7], which has been shown to reduce Hospital-Acquired Pressure Injury (HAPIs) by increasing adherence to turn protocols and is further described later in the paper, is a cost-effective prevention strategy when used as an adjunct to standard care. The analysis expected cost savings of $6,621 per patient over a 1-year period, with the mean cost to treat a HAPI was estimated at $21,767 per occurrence [8].

Furthermore, WDs' non-invasiveness spares the patient from the need for invasive monitoring, leading to increased patient satisfaction and mobility while reducing pain and adverse events. This could apply to patients admitted to ICU following complex or complicated surgery without need for invasive monitoring and to patients recovering from acute critical illness with increased mobility and out-of-bed physical therapy sessions. WDs can also fill the gap between high monitoring wards, such as the ICU, and standard wards. They can be employed in step-down units, which are often the location of choice for patients requiring an intermediate level of care between the invasiveness and monitoring of ICU and the low level of monitoring of standard wards. Even more importantly, wearable devices could bring intermediate level monitoring into standard wards, enabling continuous observation and improving patient safety even in less resource-intensive settings. This is even more clear in pediatric and neonatal populations. In infants and children, although continuous monitoring is essential in pediatric and neonatal ICU, the presence of electrodes, hardware and wires impedes skin-to-skin contact between parents and infants, complicating feeding and diaper changes, and causing frustration for both parents and healthcare providers. Additionally, invasive monitoring in pediatric patients necessitates additional training due to patient size and the use of smaller catheters, which could present a higher risk of positioning complications compared to the settings used in adults. A wireless WD could therefore represent an advantage in this setting.

The present paper is a scoping literature review of current, high-quality articles in the field of wearable devices, focusing on those which have been tested in the ICU and critical settings. Information is obtained from scientific journals in various fields, such as biomedical engineering, digital health and intensive care medicine. The articles were searched on Google Scholar, PubMed, Scopus, IEEEXplore, and Web of Science, using keywords such as “intensive care unit” (“ICU”), “non-invasive measurements”, “wearable devices”. Table 1 summarizes the available technologies and parameters that can be measured.

**Table 1:** Available technologies found in WDs and parameters that can be measured

Technology	Cardiac parameters	Respiratory parameters	Hemodynamics	Temperature	Other parameters
Electrocardiography (ECG) electrodes	Heart rate, heart rate variability, waveforms of the P-QRS-T complex	Respiratory rate (static)	Arterial blood pressure (in combination with PPG)		
Photoplethysmography (PPG) sensor	Pulse rate, pulse rate variability	Respiratory rate (static), Peripheral blood oxygen saturation,	Arterial blood pressure (in combination with ECG/SCG)		
Inertial sensors	Heart rate, heart rate variability	Respiratory rate, tidal volume	Arterial blood pressure (in combination with PPG)		Posture/position, human activity recognition
Piezoresistive sensors		Respiratory rate, tidal volume			
Capacitive sensors		Respiratory rate			
Respiratory inductance plethysmography (RIP)		Respiratory rate			
Electrical impedance tomography (EIT)		Respiratory rate, ventilation, pulmonary imaging			
Fiber-optic sensors		Respiratory rate		Body temperature	
Acoustic sensors	Heart rate, heart sounds	Respiratory rate, respiratory sounds			
Electromyography (EMG) electrodes		Activity of the muscles of the chest wall			
Electrochemical sensors					Partial pressure of transcutaneous carbon dioxide, blood glucose (subcutaneous)
Resistive temperature detector (RTD)				Body temperature	
Sweat sensors					Blood glucose
Barometers					Human activity recognition
Electroencephalography (EEG) electrodes					Brain activity at different wavelengths

**Fig. 1:**
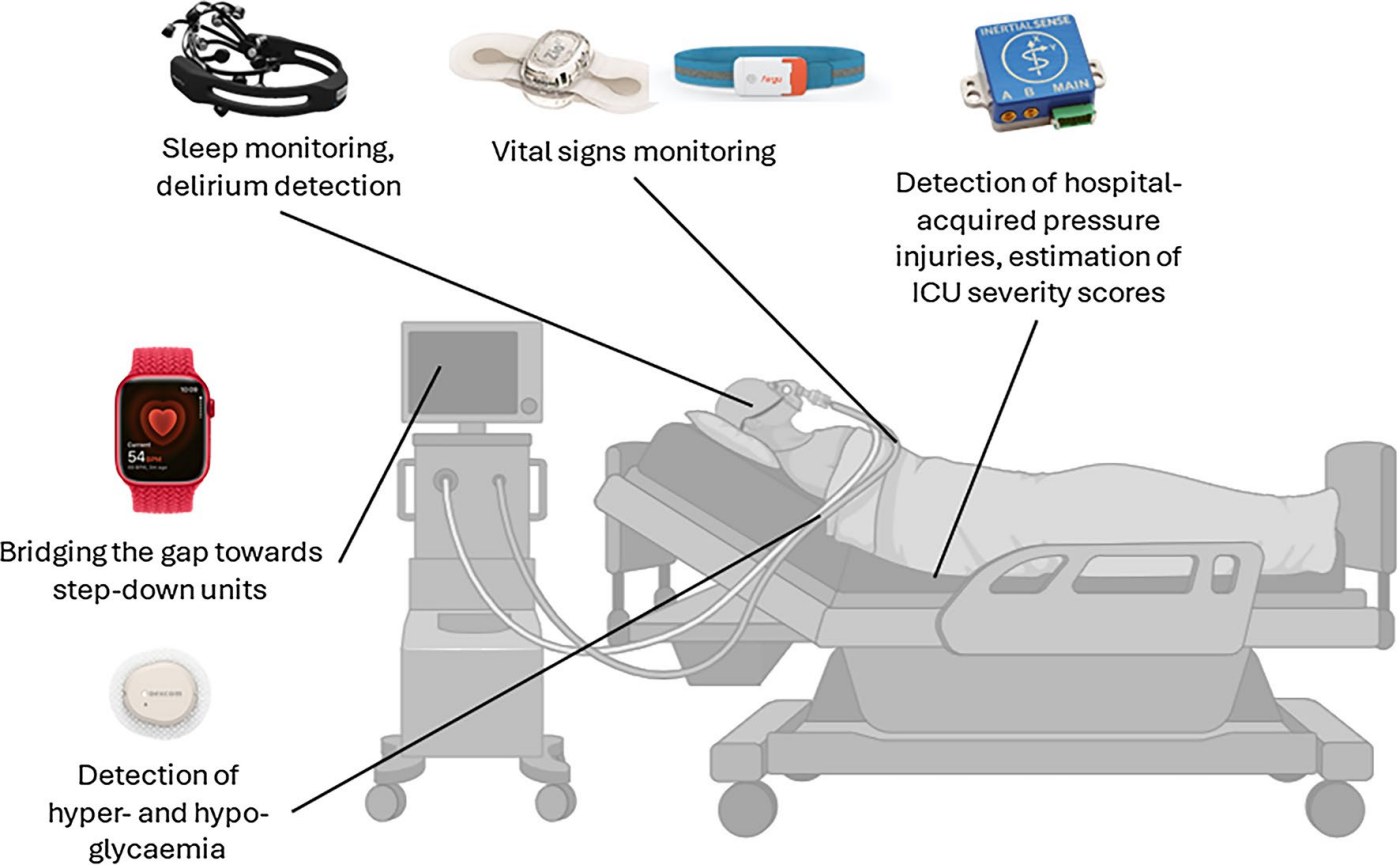
Use of wearable devices in the ICU: applications and technologies used. Vital signs monitoring: cardiac and respiratory monitor; sleep monitoring and delirium detection: wearable EEG; detection of hyper- and hypoglycemia: continuous glucose monitor;  detection of hospital-acquired pressure injuries and estimation of ICU severity scores: inertial measurement units; bridging the gap towards step down units: low-cost devices

Figure 1 illustrates some applications where use of WDs has been documented in the scientific literature.

In the following sections, we presents the available techniques to monitor a specific organ or physiological function, followed by the possible clinical applications.

## Cardiac function

Techniques used in WDs to measure cardiac function include electrocardiography (ECG), photoplethysmography (PPG), seismocardiography (SCG) [9], and phonocardiography (PCG).

### Electrocardiography

ECG consists in the noninvasive recording of the electrical activity of the heart and is performed by placing electrodes to detect abnormalities [10]. The parameters that are usually extracted are heart rate (HR), HR variability (HRV), and the waveforms of the P-QRS-T complex. Changing heart rhythms can only be detected in long-term recordings with continuous ECG (cECG), and a possible alternative to traditional equipment is represented by patch ECG devices [11]. Such devices are in fact unobtrusive, wire free, and able to record from weeks to months. Examples are the Next-generation Zio^®^, Zio^®^ XT and Zio^®^ AT patch monitors (iRhythm Technologies Inc., San Francisco, CA, USA) [12] and the KardiaPatch monitor (AliveCor Inc., Mountain View, CA, USA) [13]. Textile electrodes are an alternative that is particularly interesting because of the increased comfort of long-term recordings. Most e-textile systems are based on two and occasionally more leads to recording the ECG. There are several garments present on the market which can perform multiple-lead ECG, for instance the Healer TeleHealth System (L.I.F.E. Italia Srl, Milan, Italy) [14].

A Vietnamese research team demonstrated the feasibility of employing affordable WDs for continuous monitoring of vital signs in a LMIC. The monitored signals included HR, pulse rate (PR), and peripheral blood oxygen saturation (SpO_2_) in critically ill patients with tetanus, using medical-grade devices capable of exporting continuous waveform data. This study used a single-channel patch ECG (ePatch V.1.0, BioTelemetry, USA) and a wrist-worn pulse oximeter based on a fingertip PPG sensor (SmartCare Analytics, UK) [4]. In another study conducted in Rwanda, the investigators employed the VitalPatch® RTM (VitalConnect Inc., San Jose, CA, USA) [15], which is a single-lead ECG equipped with an accelerometer. The authors used the device for the continuous monitoring of HR, respiratory rate (RR), and body temperature in septic patients admitted to the Emergency Department. The study demonstrated the device’s suitability and accuracy for the intended use. There were nine significant vital signs changes in the enrolled population, and the WD detected them an average of 5.5 h earlier than standard intermittent monitoring, highlighting its potential utility in resource-constrained settings [16].

### Seismocardiography

SCG is the process of measuring the vibrations of the body, specifically those occurring in the thoracic region, which are induced by the contractions of the heart and the ejection of blood from the ventricles [17]. It is possible to record a seismocardiogram by placing an Inertial Measurement Unit (IMU) on the chest of a person. The parameters that can be obtained from SCG are HR and HRV. The main drawback of this strategy is that it is highly sensitive to noncardiac movements, so it is indicated only in static conditions, which can be often found in the context of the ICU. Further advancements in movement artifact removal algorithms could enable reliable use of seismocardiography during patient mobilization, unlocking additional benefits. A study conducted in a newborn and pediatric population [18] assessed a soft skin-interfaced biosensor employing advanced wireless technology for the monitoring of several vital parameters in neonatal and pediatric ICUs. Specifically, two units were used, *i.e.*, a unit on the chest equipped with an ECG for HR measurement, a three-axis accelerometer from which SCG is derived, and a body temperature sensor, and a unit on the foot equipped with a PPG sensor for PR and SpO_2_ monitoring.

### Photoplethysmography

PPG is an optical technique utilized to detect changes in blood flow volume within a peripheral vascular bed [19, 20]. PPG measurement sites include fingertip, wrist (as in smartwatches and personal fitness trackers, or PFTs [21]), arm, earlobe, esophagus, forehead, thigh, leg, and ankle. The two main parameters measured by PPG are PR, serving as a surrogate for HR [22, 23], and SpO_2_, which we further discuss in the section dedicated to respiratory monitoring. Early accuracy studies conducted in ICU demonstrated that it is feasible to use PPG-based fitness trackers to assess PR [27]. A wrist-worn commercial PFT (Fitbit Charge HR, Fitbit, San Francisco, California, USA) was employed in 50 stable ICU patients for detection of PR, activity, and sleep. Out of 12,358 readings paired from cECG and PFT data, the median difference between PFT-derived HR and cECG-derived HR was 1 beat per minute, with 73% of the readings within 5 beats per minute of the cECG values. The device was reliable and well tolerated, but its sensitivity for the detection of tachycardia was moderate, also considering the mild severity in this population.

Discharge from critical illness necessitates supervision in environments with reduced monitoring capabilities. One of the most innovative possibilities raised by WDs is to be employed as monitoring system after discharge from ICU in step-down units or regular wards. In an early study following WD application after recovery from critical illness, Kroll et al. [28] compared PR and sleep data obtained from the Fitbit Charge PR with data derived from telemetry and sleep questionnaires. In this study, the high specificity (98.8%) but low to moderate sensitivity (69.5%) identified for the detection of tachycardia suggests that wearable-derived PR tracking would be highly specific, thereby mitigating alarm fatigue, but may lack sensitivity in some situations, resulting in missed detection of PR excursions. This highlights that wearable data processing algorithms should be further investigated before their clinical deployment, but also underlines how intermediate and step-down unit could be the best setting to start clinical use of these monitoring systems.

### Phonocardiography

Acoustic sensors, such as microphones and piezoresistive sensors, are used to detect heart sounds in PCG [29]. Wearables equipped with such sensors are capable of detecting heart sounds like S1 (closure of atrioventricular valves) and S2 (closure of semilunar valves), and possibly capture additional sounds (S3, S4) or even murmurs. PCG and ECG can be used in combination: this technique is called electro-phonocardiography and can be used in wearables due to its non-invasiveness [30].

### Arterial blood pressure measurement

Arterial blood pressure (ABP) is another parameter of interest in cardiac monitoring. Meizoso and colleagues analyzed the application of a miniature wireless vital signs monitor (WVSM^®^, Athena GTX, Des Moines, Iowa, USA) [31] for triaging trauma patients [32]. Even if the device was initially developed for battlefields, there was good correlation between systolic blood pressure (BP) and HR measured with the device and gold-standard ICU monitoring (standard ECG and cuff non-invasive blood pressure measurement). Nonetheless, when employed to identify the most severe conditions, the device could not accurately classify the most critical patients.

ABP can now be measured noninvasively by wearable cuffless devices. Cuffless methods to perform this measurement exploit either the pulse arrival time (PAT) or the pulse transit time (PTT). PAT is the time that the pulse waveform takes to go from the heart to a distal site, and is obtained by computing the temporal difference between the R-peak in the ECG signal or the beat detected in the SCG signal and the peak of the PPG waveform. PTT, on the other hand, is the time that the pulse waveform takes to go from a proximal to a distal site, so it can be computed by measuring the temporal difference occurring between the same peak of the PPG signal in two different sites [33]. A drawback of this method is that initial calibration is required, therefore the quality of the final measurement is highly dependent on this procedure [33]. Another limitation is its accuracy, which is still lower than what is needed for clinical use [34]. These solutions retain a great potential in the future, with expected improvements in accuracy and reliability.

The most known reference for the validation of cuffless BP devices is the IEEE standard (published in 2014, with an amendment in 2019), which sets the number of participants in a validation study, the type of validation protocol, and the error with respect to a gold standard method [35, 36]. This standard proposes a validation method for intermittent measurements, not for continuous monitoring [37]. Furthermore, there has been an ongoing initiative to establish new standards for validating continuous measurements, such as the ISO 81060-3:2022 (non-invasive sphygmomanometers, part 3: clinical investigation of continuous automated measurement type) [38].

## Respiration

There are several approaches used in respiratory signal monitoring with WDs: estimations derived from other waveforms, from chest wall movements, and from changes in the surrounding environment. In addition to the respiratory signal, monitoring of blood gases concentration is relevant to assess the gas exchange function of the respiratory system.

### Respiratory waveforms derived from cardiac signal

The ECG and the PPG waveforms allow to derive the RR. Chest movements affecting the electrodes cause morphological changes to the shape of the QRS complex of the ECG [39]. The HealthPatch MD (VitalConnect, San Jose, California, USA) is an example of RR derived by the ECG, specifically RR is derived from the combined information from three sources: an embedded algorithm uses a weighted average of two characteristics of the ECG signal, *i.e.*, QRS amplitude modulation and respiratory sinus arrhythmia, and accelerometer data produced by chest movement during respiration [40]. Breteler et al. compared data from this wearable with an ICU monitor, also considering RR < 8 bpm and RR > 30 bpm, and obtained a bias of 4.4 bpm and limits of agreement [− 5.8; + 14.7] bpm [41]. Such results are not satisfactory for ICU applications and highlight the limits of ECG waveform analysis for RR estimation. Similarly, the PPG signal can also be used to derive an estimation of RR because of respiration-induced variations in frequency, intensity and amplitude of the PPG waveform [42].

### Chest wall movements

Chest wall movements can be detected by several different sensors, which can be based on changes in resistance, capacitance, inductance, impedance, inertial measurements, light, sound [43]. From chest wall movements, it is also possible to derive RR with good accuracy, and there is increasing research interest in the estimation of tidal volume (V_T_) from data that can be acquired with WDs [44].

Piezoresistive sensors allow detection of chest wall movements by changes in resistance, and several garments are based on this technology [45], including those produced by L.I.F.E. Italia Srl [14] and those used in the MyHeart project [46] and the ProeTEX project [47]. These sensor-equipped garments are particularly suitable for patients in step-down units or standard wards. However, their application can be challenging in bedridden patients in ICUs or after thoracic or abdominal surgery, due to drainages and wound care. An alternative consists in using chest-worn bands, for instance the AirGo™ device (myAirgo, Milan, Italy) is a thoracic band which measures the abdominal rib cage circumference changes by means of a resistive sensor and derives RR from circumference changes data [48–50]. Band-Aid-like devices exploiting piezoresistive sensors have laso been described [51]. Compared to garment related technology, the thoracic band can be more easily applied to ICU and bedridden patients.

Capacitive sensors measure the capacitance between two electrodes. If the two electrodes are placed on the chest wall, the latter acts as a dielectric material, and the variations in capacitance will correlate with RR [52]. The chest wall can alternatively be considered an electrode itself; therefore the capacitance is between the electrode attached to the skin and the conductive body fluid [53].

Respiratory inductance plethysmography (RIP) is based on changes in inductance; two transducer bands are placed around the chest wall to monitor excursions of the thorax and abdomen [54]. The sensorized garments Hexoskin and Astroskin (Carré Technologies, Montréal, Canada) are examples of devices based on RIP [55]. The latter were developed for space research and were largely studied in ambulatory patients more than in critically ill and ICU patients.

Impedance pneumography consists in the recording of the bioimpedance of the chest wall for the indirect measurement of respiration, using superficial electrodes [56]. Breteler et al. [41] compared the Sensium^®^ Wireless Vitals Monitoring patch (TSC Connected Care, Abingdon, United Kingdom), an impedance pneumography device, in a population of critically ill patients. The device was compared with the standard ICU monitor in a wide range of values, including RR < 8 bpm and RR < 30 bpm; resulting in a bias of − 0.8 bpm with [-8.5; + 6.9] bpm as agreement limits.

Electrical impedance tomography (EIT) is an imaging technique that also exploits the changes in impedance of the chest wall during breathing, exploiting the same principle as impedance tomography, and consists in applying a current or voltage pattern through electrodes placed on the chest wall and reconstructing the internal conductivity distribution using the collected voltages from the electrodes [57]. In addition to RR and ventilation, pulmonary imaging is one of the main applications of EIT, which is an application of great interest in the ICU because it can be used at the patient’s bed, without transfers to dedicated rooms, and in a continuous way due to its non-invasiveness. There have been research efforts towards the realization of wearable EIT solutions, such as the work by Oh et al. [58] and the work by Hu et al. [59]. EIT wearability would allow to mobilize patients in the ICU, yet technological challenges remain, specifically battery size and weight.

IMUs can also be used to monitor respiratory parameters like RR and V_T_ [60]. Generally, one or two IMUs are placed on the chest wall [61], but there are also cases where one IMU is placed on the nasal septum [62, 63] or on the auricle [64]. A commercial example with one IMU is the MonBaby monitor (MonDevices, New York, NY, USA), which detects breathing movements and changes in the position of infants, thus combining two important functions needed in the ICU, as it is later described in the subsection dedicated to “Position monitoring”. A configuration with three IMU-based units can also be implemented [65], and provides more detailed information on chest wall movement. Two units, of which one is on the thorax and the other is on the abdomen, detect respiration; the third unit is placed on a reference point not involved in respiratory movement which controls position and activity [66–68]. This specific configuration has two degrees of freedom as it can detect the movement of the thorax separately from the movement of the abdomen; this could be used in the future to identify paradoxical respiration, which is very difficult to detect with standard ICU monitor.

Fiber-optic sensors can be used for respiratory monitoring because chest wall displacement affects fiber-optic signals. Shape changes and bending produced by inspiration cause shift in the light intensity exiting the fiber, and these changes are connected to respiratory movement [69].

Finally, electromyography (EMG) devices detect the electrical signals present in muscles [70]. Surface electromyography (sEMG), a non-invasive method for recording muscle activity through electrodes placed on the skin, can be performed using wearable devices such as armbands or patches for convenient and continuous monitoring. sEMG can be used to detect the presence of activity in respiratory muscles to assess the effectiveness of ventilatory efforts, for instance in combination with invasive ventilation.

### Changes in the surrounding environment

Acoustic sensors positioned within the ear canal, trachea, and nasal regions can be employed for respiratory sounds detections and recording. After the sound is recorded, spectrum analysis is performed to detect RR [71]. This is exploited by the Radius-7^™^ (Masimo Corporation, Irvine, CA, USA) monitor, which is connected to acoustic adhesive sensor applied on the neck (RRa^®^). The study by Breteler et al. [41], which we already reported for techniques using ECG waveform modulation and impedance pneumography, also reports the comparison of the RRa^®^ acoustic sensor with the ICU bedsite monitor, with a bias of 0.2 bpm and limits of agreement [− 6.6; + 6.3] bpm. These results were obtained in a wide range of RR values, which is representative of the conditions typically encountered in the ICU population.

### Concentration of gases in the blood

Other parameters of interest in respiratory medicine are the concentrations of gases in the blood, mainly the partial pressure of arterial O_2_ and the partial pressure of arterial CO_2_ (PaCO_2_). SpO_2_ is commonly used as a surrogate of PaO_2_ by means of PPG devices, generally at the fingertip. This technology is widely accepted in clinical practice because of the established mathematical relationship between SpO_2_ and PaO_2_, known in literature as the oxygen dissociation curve and largely studied in the past century [72].

Conversely, PaCO_2_ is obtained with invasive measurements but is known to be correlated with the non-invasive partial pressure of transcutaneous CO_2_ (PtCO_2_) in static conditions [73]. CO_2_ gas diffuses through body tissue and skin; transcutaneous measurements exploit this by using a sensor (generally electrochemical or optical) placed on the skin surface [20, 74]. WDs for the measurement of PtCO_2_ are not commercially available but they are a promising field of research [75].

## Body temperature

WD can be used to monitor body temperature [76]. Some of the previously mentioned garments embed sensors for temperature monitoring, for instance the Astroskin garment [9], and the employed solutions in case of garments are temperature sensing fabrics. The most common are resistive temperature detectors (RTDs) and Fiber Bragg Grating (FBG) sensors integrated into the fabric [45]. RTDs change their resistance with changing temperatures, while the FBG sensor is an optical fiber with a core that includes a periodic section made of a material with a different refractive index. As light propagates within the fiber, certain wavelengths are absorbed, while others pass through. The light that returns exhibits a wavelength shift indicative of the skin’s temperature [77]. Smartwatches often include temperature sensors but is not always specified whether it is body or environmental temperature. Socks for foot temperature monitoring have been developed for diabetic patients, [78] but they could find application in the ICU too, specifically in case of vascular deficiency and insufficient peripheral perfusion, such as severe septic shock requiring high dose vasopressors or after vascular surgery.

## Blood glucose concentration

Glycemic control is an important aspect of ICU care, as critically ill patients commonly experience insulin resistance and episodes of hypoglycemia and hyperglycemia. Hyperglycemia episodes are associated with mortality and morbidity, while achieving tight glycemic control is nonetheless challenging due to the danger of hypoglycemia, as highlighted in the NICE-SUGAR study [79].

Current blood glucose (BG) measurement methods involve point-of-care glucose meters and blood gas analysis. Such technologies can be time-consuming for the ICU staff and at the same time painful for patients as blood samples are collected. Since these methods are not continuous, another issue is that events occurring between readings are often missed.

The frequent measures available from continuous glucose monitoring (CGM) devices offer significant opportunity to monitor and improve the safety and performance of glucose monitoring and control in critically ill patients without impacting staff workload [80]. CGM allows more rapid treatment responses to highly dynamic changes in patient condition and glycemia, by enabling insulin adjustments in accordance with guidelines and nutritional needs. This has proven particularly useful in periods of staff strain, such as during the COVID-19 pandemic. In COVID-19 critically ill patients, CGM systems demonstrated reliability and accuracy in monitoring blood glucose levels [81]. Moreover, CGM can diminish the frequency of invasive blood collection, mitigating the risk of iatrogenic anemia, or of fingerstick glucose monitoring, reducing patient discomfort. However, CGM devices can present large accuracy errors due to sensor drift, bias, and noise [82].

There are two types of CGM used in the ICU: intra-vascular CGM (IV-CGM) and subcutaneous CGM (SC-CGM). As SC-CGM uses an electrochemical sensor to measure glucose levels every minute through glucose oxidase. SC-CGM are inserted in the adipose tissue, and commercial models last about 2 weeks without requiring changes. There are several SC-CGM available, many of which have the capability to notify the healthcare provider of critically low or high measures, such as FreeStyle Libre 3 (Abbott Laboratories, Abbott Park, IL, US) [83], Dexcom G7 (DexCom Inc., San Diego, CA, US) [84], and Guardian™ (Medtronic plc, Dublin, Ireland) [85]. There is comparable accuracy between of IV-CGM and SC-CGM in some setting, therefore lowering the demand for invasive sampling and devices [86], even if the time-lag between adipose tissue and blood should be considered. [88]. In the overnight fasting state in healthy adults, the physiological delay of glucose transport from the vascular to the interstitial space was found to be a non-clinically significant 5–6 min [87]. Nonetheless, time can be higher in patients with shock conditions under vasopressors, and this should be further studied before application in the acute phase of critical care [89]. Future devices may be capable of monitoring glucose levels using sweat analysis [90], even if this has not yet been examined in acute or critically ill patients.

## Position

HAPIs are insidious, multi-factorial complications arising from sustained pressure and/or damage caused by shear and friction forces. Joint prevention guidelines from the European, American, and Pan-Pacific agencies for preventing pressure injuries recommend individualized care plans, appropriate supportive surfaces, and modulating the frequency of repositioning based on individual patient needs. The standard of care for patients in the ICU is redistribution of weight every 2 h while in bed (with a minimum turn angle of 20°), and every hour while in a chair. However, globally, studies evaluating patient turning reveal poor compliance with turning protocols, and varying rates of pressure injuries (3–37%) [91].

Position can be easily monitored with IMUs, which can be used to determine spatial orientation depending on the axis where gravity acceleration is sensed, and to understand whether and when position changes.

The LEAF Patient Monitoring System™ (Smith + Nephew, Inc., Watford, UK) [7] is an example of a wearable designed to be placed on the chest using adhesive patches, allowing patients mobilization while wearing the device. It assesses the patient’s body position, aiding in the prevention of pressure injuries. This technology assists clinical staff by providing information on patients who require assistance in turning and indicating when repositioning is necessary. Data from the device is sent wirelessly to a central monitoring station and updated on a display every 10 s to help healthcare providers identify patients who need turning.

In a randomized controlled trial by Pickham et al. [91], patients were assigned to receive either standard care to mitigate HAPIs or intervention utilizing optimal practices informed by real-time data from the LEAF Patient Monitoring System™. Nurses attending patients in the treatment group would get visual alerts if the patient was not repositioned in accordance with local guidelines. The study showed that the group monitored with the WD had one third of the HAPIs during ICU admission with respect to the control group ([0.7 vs 2.3%], p = 0.031), demonstrating that a simple application could be very effective and easily implemented in the ICU.

Another study [92] demonstrated an increase in repositioning compliance by nursing on residents of a nursing home, where nurses have been instructed to use the LEAF Patient Monitoring System^™^. Compared to a 3 day baseline period, with a 61.4% repositioning compliance, the repositioning compliance increased to 81.5% on average during the 18-day intervention.

## Activity and movement

Early mobilization in ICU patients leads to fewer physical injuries, reduced duration on a mechanical ventilator, and decreased length of hospital stay.

PFTs, also called activity trackers, generally include human activity recognition (HAR) algorithms, which can be based on different types of sensors [93]. IMUs represent the most common strategy to monitor activity, and they are generally composed of accelerometers, gyroscopes, and magnetometers. Accelerometers demonstrate a great capability for HAR and can be combined with gyroscopes to improve performance in fall detection, gait analysis and HAR. Magnetometers can also be used to improve HAR, however, they suffer from environmental disturbances and thus are often discarded. Barometric pressure sensors can be useful in HAR, especially to detect falls and to recognize whether a person is climbing stairs [94]. Smartphones can be used as activity trackers as they generally have embedded IMUs [95].

Step counting is one of the major features of PFTs, but the performance of step counting algorithms varies among devices. In a study conducted in 2015 [96], a strong correlation was identified in step counts when comparing measurements obtained from Fitbit devices with those obtained using gold-standard methods. However, the study revealed lower accuracy specifically at slow walking paces, which are commonly observed among older adults [97] and expected among ICU patients. Step counting devices can be more useful in intermediate care settings and standard wards.

On the contrary, HAR systems can be used to target patient mobilization by enabling individualized monitoring of mobility levels and activity patterns. A scientific expert panel's recommendations [98] focused on tailored positioning and mobilization protocols. HAR-enabled PFTs can report the real level of daily physical activity, providing actionable data and ensuring timely adjustment of physical therapy while adhering to safety protocols.

Movement data could also assist in assessing critical illness severity status, especially in conjunction with clinical data from electronic health records (EHR). One of the main issues with traditional ICU severity score systems like SOFA and APACHE II is that they use either static variable threshold, such as fixed thresholds for physiological variables which may not accurately reflect the dynamic changing of clinical conditions, or additive scores. In fact, additive scores include several variables by simple addition or weighting of parameters, but this is an oversimplification of the complex interactions happening in the human body. Consequently, these scores have limited predictive accuracy. Most of these scores are human-dependent, and can be compiled only at fixed time intervals. In a prospective study, wrist-worn accelerometers were combined with clinical data from EHR to estimate patient acuity, from combining movement information and vital parameters [99]. While the idea of developing an electronic phenotype of acuity from movement data (*i.e.*, stable or unstable) was proposed in a previous work [100], Sena et al. [99] employed deep learning models for time-series accelerometric data, integrating clinical features to predict patient acuity states with a focus on models like visual geometry groups, ResNet, and custom transformer-based networks. Accelerometers were used to assess patients' physical movements, capturing 9286 h of data, excluding patients ready to be discharged or unable to consent. The authors of the study report results of the deep learning models used on different combinations of data; the best results were obtained with a ResNet model with clinical, demographic, and accelerometer data as input (area under the curve = 0.73, precision = 0.80, sensitivity = 0.60, specificity = 0.79, f1-score = 0.77).

Accelerometers were among the first devices tested as a non-invasive method to assess sleep quality in critically ill patients, who often experience significant sleep disturbances due to their medical conditions and the ICU environment, where traditional sleep assessment methods, such as polysomnography, are unfeasible. This evaluation, called actigraphy, was summarized in a recent review on the quality of sleep in intensive care, including results from nine studies [101]. In most studies the actigraph was placed on the wrist, with two studies also assessing ankles. Only five [102–106] of the nine studies analyzed in the review evaluated actigraphy measurements against a reference. In general sample sizes were small, and often data were collected for only one night. Therefore, further research on actigraphy in critically ill patients is needed before it can be used in this population, with larger sample sizes, longer monitoring durations, and specific sensors and software tested for critically ill individuals who often have low mobility states and often receive sedatives and analgesics.

Another field of interest is delirium, which is an acute disturbance in cognition, characterized by confusion with inattention, disorganized thinking, and altered levels of consciousness [107]. Delirium is common in intensive care and in general in elderly hospitalized acute patients. The clinical course is often fluctuating, ranging from hypokinetic to hyperkinetic manifestation. Apart from overt agitation cognitive alteration, delirium is difficult to detect unless specifically screened for by standardized testing. New technologies assessing delirium by movement detection using cameras and artificial intelligence (AI) or accelerometers are gaining traction as a promising approach. Changes in physical activity, especially during crucial times like overnight, may indicate the onset or severity of delirium. Lehmkuhl et al. [108] conducted an observational study in mixed medical/surgical ICUs, assessing body posture in 39 mechanically ventilated patients utilizing a thigh- and chest-mounted accelerometer during their ICU stay. They emphasized the role of physical activity, particularly in the evening, related with nursing interventions designed to sustain the natural circadian rhythm which is essential for preventing delirium. Neerland et al. [109] are conducting multicentric randomized trial on pharmacological therapy for delirium prevention. They are employing body-worn accelerometers to provide insight into motor activity patterns in different subtypes of delirium and compare validated scales including Confusion Assessment Method (CAM)-ICU-7 [110], Richmond Agitation-Sedation Scale (RASS) [111] and Montral Cognitive Assessment (MoCA) [112]. Similar findings were confirmed by Davoudi et al. [113], which demonstrated that monitoring of patients (facial expressions, extremity movements, and postures) by means of WDs and contactless monitoring of the surrounding environment is achievable, and resulting data can be associated with delirium, even if this has not been examined in the general ICU population but in hospitalized patients and hospice patients.

## Brain activity

Electroencephalography (EEG) is a technique designed to monitor brain activity by recording the electrical signals non-invasively and is being increasingly used to implement brain–computer interfaces. The biopotentials are detected using surface electrodes placed on the scalp [45]. EEG detects wavelengths < 80 Hz, and the waves that are generated by brain activity are classified as delta (1–4 Hz), theta (4–8 Hz), alpha (8–12 Hz), beta (12–25 Hz), and gamma (> 25 Hz).

Conventional, wired EEG systems are not user-friendly for real-life applications, but recent advancements in wireless EEG systems have allowed for drastic improvement of device portability, with the goal of providing more user flexibility [114]. Wireless transmitters in EEG systems play a crucial role in enabling the portability and flexibility of brain activity monitoring. These transmitters are embedded within the EEG system and are responsible for capturing the electrical signals generated by neurons firing in the brain. Once these signals are detected by electrodes placed on the scalp, the transmitter converts the analog electrical signals into digital data and sends them wirelessly, via Bluetooth or Wi-Fi to a nearby device such as a computer, smartphone, or tablet for real-time analysis. EEG systems may be equipped with wet or dry electrodes, varying the number of channels depending on the form of the EEG device (such as caps [115], headband, headsets, visor, smart glasses [116]).

Delirium has long been recognized as connected with sleep deprivation and alteration in sleep–wake patterns in ICU patients [117], and in non-rapid eye movement (nREM) phases of sleep. Wearable EEG could help to detect, characterize and target this condition [118]. Specifically, wearables may be utilized to monitor methods aimed at enhancing sleep–wake patterns in patients, as well as monitoring effects of lighting and noise levels in the ICU setting. EEG-monitoring wearable devices may represent an affordable solution to track NREM and in quality and depth of sleep in the ICU [119].

## Discussion

WDs are becoming more and more common in various application settings, with a subsequent reduction in retail prices, which extend well beyond critical care. The authors were able to test perioperative risk stratification using a simple commercial device with a cost below 80 euros [22, 23]. Low retail prices facilitate the availability of an extensive monitoring platform at a relatively low expense. Moreover, as these devices are often non-invasive, the quantity and cost of disposables associated with their usage are limited. Devices can be utilized multiple consecutive times, again significantly diminishing costs. Additionally, we should consider indirect costs. WD low invasiveness levels can reduce costs associated with complications and infections. Simplicity and automation could offer various advantages to healthcare personnel, such as freeing up time for improved patient care and optimizing resource utilization. The capacity of wearables to gather and relay data instantaneously may also improve clinical decision-making, resulting in more prompt and efficient therapies.

The clinical applicability regarding the use of WDs in healthcare settings has emerged only in recent years, due to the complexity of critical illness and the strict regulatory requirements for accuracy and safety of medical devices.

Data validation represents a relevant issue in the field of WDs in the ICU setting. Most validation studies derive from community environments involving healthy adults, often outside the hospital setting. The reliability of data obtained from unstable or critically ill patients is still debated [40]. Research demonstrates a significant association between WD-derived HR measurements with nurses’ manual observations and standard medical monitoring, but this association is less significant when comparing RR measurements. Medical-grade wearable patches in surgical patients yielded similar results when compared to the gold standard from ICU monitors. The suitability of low-cost wearables to record high resolution waveform data is even less certain, as these systems may be limited by artifacts, noise and interference, movement artefact, and missing data [4].

Another issue of WDs in the ICU is the significant amount of data and signal loss. Multiple factors contribute to this issue, including varying quality of devices, inadequate Wi-Fi and cellular connectivity due to the high number of connected devices in a relatively small space. Other factors that contribute to this issue are situations related to clinical environment, such as patients with seizure or shivering, frequent patient mobilization, or need for prone positioning and other contextual issues. Van et al. [4], who tested the use of wearables in an ICU, discarded 7% of the wearable ECG data because they were noisy due to faulty electrodes; this was not seen in bedside monitor ECG recordings. In the same study, PPG signal loss was approximately 1% for standard monitor system, while 76% of wrist-worn WD data were unusable. SpO_2_ was the only parameter where signal loss in standard monitor data was higher than that in WD data (38 vs 26%). Patient comfort with WDs must also be also taken into consideration, specifically in patients which are unable to consistently report pressure or pain related to positioning or pressure ulcer, due to deep sedation or paralysis.

Finally, the ICU personnel now manage all ICU monitoring data and all the derived alarms. WDs and emerging technologies are expected to improve working conditions and alleviate stress only at the condition that they provide filtered and summarized data, minimizing data overload and minimizing alarm fatigue while increasing situational awareness. The large amount of data produced by WDs requires to automatically analyzed and generate alarms, possibly by means of AI, and before full application to the critical care settings it is necessary to ensure that such AI models, which are often black-box and thus not fully explainable [120], are not increasing the mental fatigue and are actually improving clinical decision making in the ICU.

## Conclusions

WDs present extraordinary new opportunities for the non-invasive collection of high-frequency data from critically ill patients. These devices are anticipated to become more and more common in critical care and acute settings, bridging the gap toward standard wards when elevated monitoring is required. While variety and volume of collected data continue to expand, further research is needed to ascertain accuracy, applicability, and reliability of WDs, as well as to implement in this field machine learning and artificial intelligence algorithms able not only to analyze complex physiological data but also to minimize data loss and poor signal quality. Despite these current limits, it is indisputable that patients will benefit from WD progress soon, moving from intermediate or step-down setting toward more complex ICU cases in the near future.

## Data Availability

Not applicable.

## Declarations

Ethics approval and consent to participate.

Not applicable.
